# Odontogenesis and neuronal differentiation characteristics of periodontal ligament stem cells from beagle dog

**DOI:** 10.1111/jcmm.15158

**Published:** 2020-03-23

**Authors:** Xiaojie Li, Dapeng Liao, Gang Sun, HanWen Chu

**Affiliations:** ^1^ Department of Dentistry Sir Run Run Shaw Hospital Zhejiang University School of Medicine Hangzhou China; ^2^ Department of Dentistry The Second Affiliated Hospital Zhejiang University School of Medicine Hangzhou China

**Keywords:** beagle dog, calcined tooth powder, neuronal differentiation, odontogenesis, periodontal ligament stem cells

## Abstract

Periodontal ligament stem cells (PDLSCs) from beagle dogs had the characteristics of multi‐directional differentiation and had great application potential in tissue engineering and cell regenerative medicine. In this study, we analysed the odontogenesis and neuronal differentiation characteristics of PDLSCs in vitro. Results showed that the calcined tooth powder (CTP) and silver nanoparticles (AgNPs) additives could induce the PDLSCs into odontogenesis differentiation; besides, the immunofluorescence staining identified that the high dosage calcined tooth powder (400 μg/mL) significantly facilitated the odontogenesis associated with BMP4 expression. While the nutritional factor (L‐glutamine, NGF (nerve growth factor), bFGF (basic fibroblast growth factor), IGF‐1 (insulin‐like growth factor‐1) and EGF (epidermal growth factor)) additives were prior to induce the PDLSCs into neuronal differentiation. Simultaneously, PDLSCs had high proliferation ability with the different supplemented additives. Importantly, the Western blot results also proved the BMP4 and SMAD1 proteins were highly expressed in the induced odontoblast, while the SOX1, NCAM1, GFAP and VEGFA proteins were all obviously expressed in the induced neurons. Hence, PDLSCs had characteristics of both odontogenesis and neuronal differentiation.

## 1 INTRODUCTION

With the rise of tissue engineering, the construction of biological substitutes such as biological tissues, organs and materials, which were used to rescue or/restore various forms of damaged or defective human tissues during recent years, has become a hotspot in the research of tissues engineering.^1^, ^2^ In endodontic treatment, there were shortcomings of the conventional pulp capping material, calcium hydroxide^3^; hence, the dental stem cells isolated from teeth and nearby tissues had been attracted more and more attention in investigating their utilities in dental prosthesis by researchers due to their self‐renewal and multilineage differentiation potential.^4^ Previous studies had showed that periodontal ligament fibroblasts (PDLFs) had great potential in maintaining and repairing the periodontal tissues.^5^, ^6^ While the PDLSCs were obtained from the beagle dog periodontal ligament (PDL) of teeth freshly extracted, which had been recognized as a useful cell source for periodontal tissue regeneration.^7^ They had the characteristics of easy to access and cryopreserved conveniently; besides, studies identified that they could induce the formation of new blood vessels and improve osteogenic.^8^, ^9^ In vitro culture studies, it had been reported that they could differentiate into odontoblasts, osteoblasts, chondrocytes and neurons ^10^, ^11^, ^12^; hence, the PDLSCs had great utility in regenerative medicine.

The calcined tooth powder (CTP) was obtained from beagle exfoliated teeth with 300°C calcine temperature, which completely removed the organic components and wiped out the antigenicity; the study of Jintao Wu, etc,^13^ had found that CTP promoted hDPSCs into osteogenic and odontogenic differentiation. While the growth factors, NGF,^14^ bFGF, EGF^15^ and IGF‐1,^16^ had been identified playing crucial roles in the differentiation of MSCs into neuronal lineage and enhance cell viability, hence, to our knowledge, in this study, we attempted to study the characteristics of PDLSCs deriving from beagle dog differentiate into odontoblasts with CTP supplementary additives; besides, the nutritional and growth factors (L‐glutamine, NGF, bFGF, IGF‐1 and EGF) were also added as supplementary additives to induce PDLSCs into neural differentiation, both of which were in the purpose of providing important insights of PDLSCs in the application of dentin/pulp tissue regeneration for future endodontic treatment.

## 2 MATERIALS AND METHODS

### 2.1 PDLSCs isolation and culture

The molars were collected from the beagle dog of 12 months old. The collected teeth were washed with PBS and obtained the periodontal membrane, then digested them in a solution of type I collagenase (3 mg/mL) (catlog: C0130, sigma) for 30‐60 minutes at 37°C; afterwards, the digestion process was inactivated with 20% foetal bovine serum (FBS). Then, the digested cell suspensions were centrifuged at 300 *g* for 10 minutes, twice to remove the tissues debris; afterwards, the PDLSCs at a density of 3.0 × 10^5^ cells were seeded in each 25 cm^2^ plate with cell culture DMEM medium supplemented with 10% foetal bovine serum, 100 U/mL penicillin and 100 U/mL streptomycin; besides, the calcined tooth powder (CTP) at a concentration of 100 and 400 μg/mL was dividedly added into the cell culture medium, and the silver nanoparticles (AgNps) (catlog: JL‐8001) 100 μmol/L concentration were also administrated into each cell culture medium.

### 2.2 Cell morphology

The PDLSCs were cultured for 7 days in 6‐well plates, and one group cells were treated with CTP (100 μg/mL) and AgNps (100 μmol/L), and the other one group cells were treated with CTP (400 μg/mL) and AgNps (100 μmol/L); the induced cell morphology was visualized at the seventh days.

### 2.3 Immunofluorescence

The PDLSCs were cultivated like above, and one group cells in each well were treated with CTP (100 μg/mL) and AgNps (100 μmol/L), and the other one group cells in each well were treated with CTP (400 μg/mL) and AgNps (100 μmol/L); after 7 days cultivation, the cells in each well were washed with PBS; then, they were fixed with 4% paraformaldehyde for 30 minutes; and afterwards, they were permeabilized with 0.25% Triton‐100 for 10 minutes at room temperature, then washed them with PBS for three times and, later, blocked them with 10% BSA for 60 minutes at 37°C. Afterwards, the cells were incubated with the primary antibody, BMP4 (catlog: ab124715, abcam) for 12 hours at 4°C. Thereafter, the cells were washed three times and incubated with the secondary antibody IgG labelled with fluorochrome (1:100; abcam) for another 60 minutes, 37°C in the cassette. Then, the cells were washed twice with PBS; subsequently, the nuclei were stained with 4,6‐diamidino‐2‐phenylindole (DAPI; 1:1000, Sigma) for 1 hour at 4°C, then washed them with PBS again and mounted the cell slide with antifade mounting medium; at last, the images of the coverslips were visualized with an inverted fluorescence microscope (Olympus).

### 2.4 Crystal violet assay

The PDLSCs were cultured for 7 days in 24‐well plates, and one group cells were treated with CTP (400 μg/mL), AgNps (100 μmol/L), and the other one group cells in each well were treated with CTP (400 μg/mL), AgNps (100 μmol/L) and the nutritional growth factors, 200 μg/mL L‐glutamine (catlog: 200‐292‐1, sigma), 10 μg/mL beta neuron growth factor 1 (NGF) (catlog: 96‐450‐01‐20, PeproTech), 10 μg/mL epidermal growth factor (EGF) (catlog: 96‐100‐47‐10, PeproTech), 10 μg/mL fibroblast growth factor (FGF) (catlog: 96‐100‐18B‐10, PeproTech) and 10 μg/mL insulin‐like growth factor 1 (IGF‐1) (catlog: 96‐100‐11‐100, PeproTech) were also all placed into the cell culture medium; then, all the cultured cells were incubated at 37°C in 5% CO_2_ incubator; besides, the cells were stained with crystal violet to visualize the cell viability at day 3, day 5 and day 7. The protocols were performed like below, the induced cells were fixed with glutaraldehyde for 20 minutes at the third day, the fifth day and the seventh day, then removed the glutaraldehyde and washed the cells with PBS for twice, afterwards, dried them in 37°C drying oven for 5 minutes and, later, the cells were stained with crystal violet for 30 minutes dyeing; thereafter, the cells in each well were dividedly received 200 µL 10% acetic acid, and after 1 hour, the cells in 24‐well plates were placed in multifunctional enzyme labelling instrument to detect the absorbance at 595 nm.

### 2.5 Cell colony formation assay

The PDLSCs were cultured for 7 days in 6‐well plates, and one group cells were treated with CTP (400 μg/mL) and AgNps (100 μmol/L), and the other one group cells were treated with CTP (400 μg/mL), AgNps (100 μmol/L) and L‐glutamine, NGF, bFGF, IGF‐1, EGF growth factors. After 7 days cultivation, the cells were washed with PBS and fixed in 4% paraformaldehyde for 30 mins; then, they were stained with crystal violet for 30 minutes at room temperature; afterwards, the number of proliferated cells was counted with Image J software; besides, all assays were performed in triplicate.

### 2.6 Western blot

The PDLSCs at a density of 3.0 × 10^5^ cells were seeded into each 25 cm^2^ plate with cell culture DMEM medium supplemented with 10% foetal bovine serum, 100 U/mL penicillin and 100 U/mL streptomycin; besides, the CTP, 400 μg/mL, and the AgNps, 100 μmol/L, concentrations were all administrated into each cell culture medium. Additionally, the nutritional growth factors, 200 μg/mL L‐glutamine, 10 μg/mL beta‐NGF, 10 μg/mL EGF, 10 μg/mL FGF and 10 μg/mL IGF‐1, were all placed into one group cell culture medium; then, all the cultured cells were incubated at 37°C in 5% CO_2_ incubator, when the cells grew well during the fifth to seventh day, washed them with PBS for three times and lysed them in RIPA buffer (Beyotime) containing 1 mmol/L protease inhibitor phenylmethanesulfonyl (PMSF). Subsequently, the proteins were denatured with hot water and measured the proteins concentration with BCA method; then, the proteins were isolated with sodium dodecyl sulfate‐polyacrylamide gel electrophoresis (SDS–PAGE); afterwards, the protein gel was transferred into polyvinylidene fluoride (PVDF; Millipore) membranes with a wet transfer apparatus (Bio‐Rad). Then, the transfered membranes were blocked in non‐fat milk for 1 hour at 4°C, later, washed the membranes with TBST for three times and, subsequently, incubated with primary antibodies, anti‐SOX1 (catlog: ab87775, abcam), anti‐BMP4 (catlog: ab39973, abcam), anti‐NCAM1 (catlog: ab133345, abcam), anti‐SMAD1 (catlog: ab33902, abcam), anti‐GFAP (catlog: ab33922, abcam) and anti‐VEGFA (catlog: ab46154, abcam) for 12 hours; besides, the GAPDH was as a control; then, the membranes were immunoblotted with anti‐rabbit IgG antibody for 1 hour. Thereafter, the protein bands were added with the chemiluminescent ECL substrate (Fermentas, MBI) and visualized them in bandscan, besides, analysed the proteins’ gray intensity with Image J Software (National Institutes of Health).

### 2.7 Statistical analysis

The differences of the data were analysed with one‐way ANOVA method in the SPSS 19.0 statistical software (IBM, Corp.). The statistically significance was *P* < .05. And, the differences displayed in graphs were analysed with Prism 6.0 software (GraphPad Software), and data were presented in mean ± standard deviation.

## 3 RESULTS

### 3.1 PDLSCs induced into odontogenesis differentiation with CTP and AgNps administration

**Figure 1:**
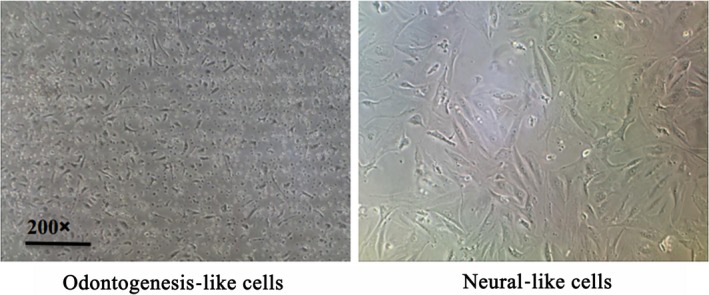
Inverted microscope detected the cell morphology of induced odontogenesis‐like cells and neural‐like cells, the induced odontogenesis‐like cells with round nucleus and small protuberance in the cell surface, while the induced neural‐like cells with obvious nucleus, sprouted cytosome like the formation of neural network

**Figure 2:**
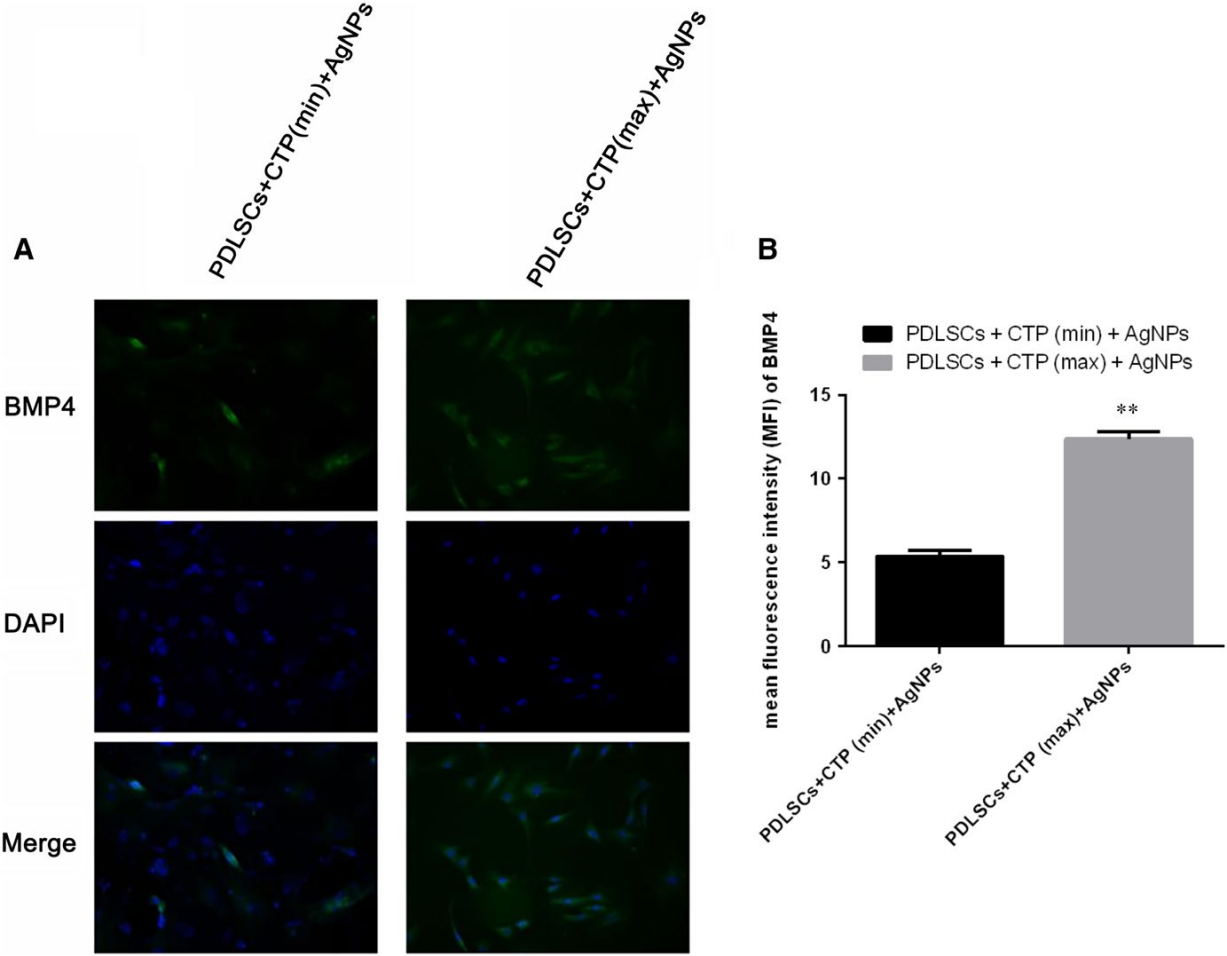
Immunofluorescence of BMP4 staining intensity in the PDLSCs cells received CTP (100 μg/mL), AgNps (100 μmol/L) induction and CTP (400 μg/mL), AgNps (100 μmol/L) administration (A). The immunofluorescence intensity standed for the relative protein expression, ^**^ *P* < .05 of protein BMP4 vs the CTP (400 μg/mL), AgNps (100 μmol/L) administration group (B)

After 7 days cultivation with CTP (100 μg/mL), AgNps (100 μmol/L) and CTP (400 μg/mL), AgNps (100 μmol/L) induction, the PDLSCs induced the odontogenesis‐like cell morphology was observed as shown in Figure 1. Besides, the immunofluorescence results showed that the high dosage CTP (400 μg/mL) facilitated the odontogenesis differentiation, for the protein BMP4 staining intensity in Figure 2, and was more obvious with CTP (400 μg/mL) and AgNps (100 μmol/L) induction than CTP (100 μg/mL) and AgNps (100 μmol/L) administration.

### 3.2 PDLSCs with high proliferation ability into odontogenesis differentiation and neuronal differentiation

**Figure 3:**
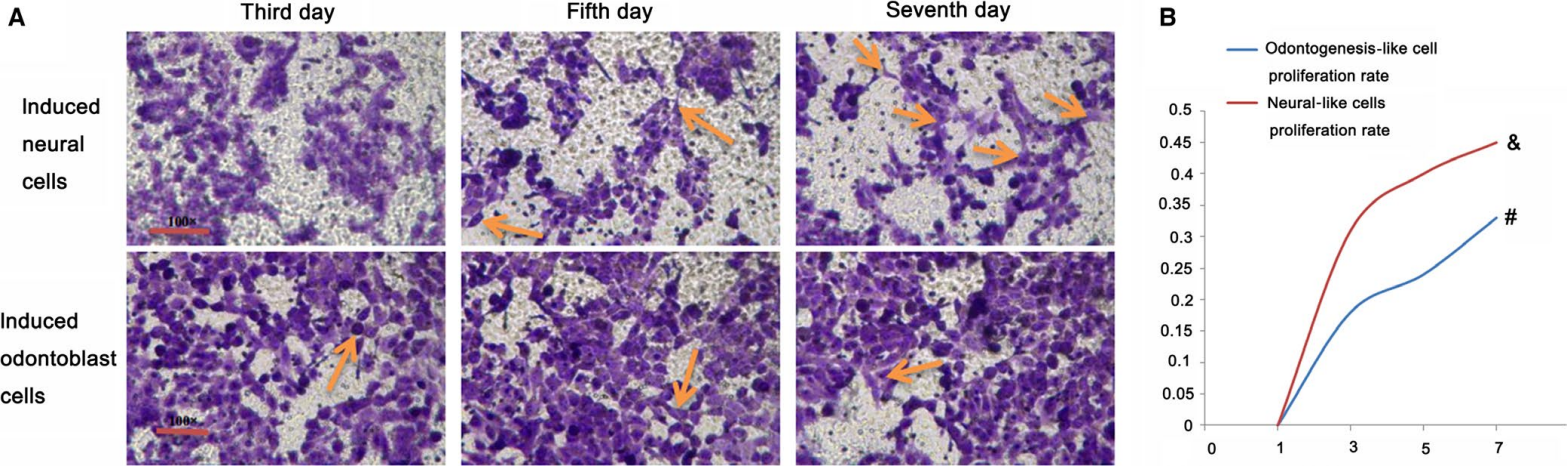
Crystal violet staining analysed the PDLSCs cell proliferation in the third day, fifth day and seventh day; the arrows pointed the induced neural‐like cells and odontogenesis‐like cells (A). The cells in logarithmic growth trend (B), ^&, #^ *P* < .05 was the significant difference of the third day vs the seventh day

**Figure 4:**
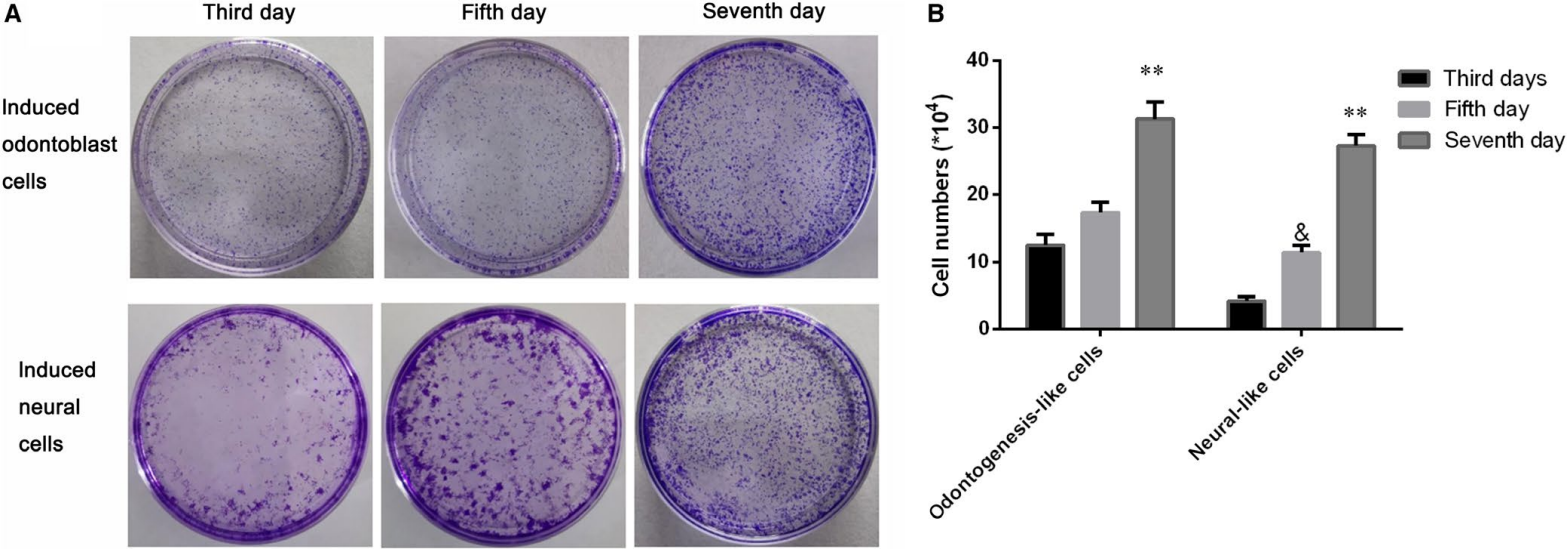
Colony formation assays showed a significant highly cell proliferation rate in both induced cells cultivated groups (A). The induced odontogenesis‐like cells and neural‐like cells with highly proliferation ability, ^**^ *P* < .05 was of significance vs the third day and fifth day, ^&^ *P* < .05 was of significance vs the third day (B)

**Figure 5:**
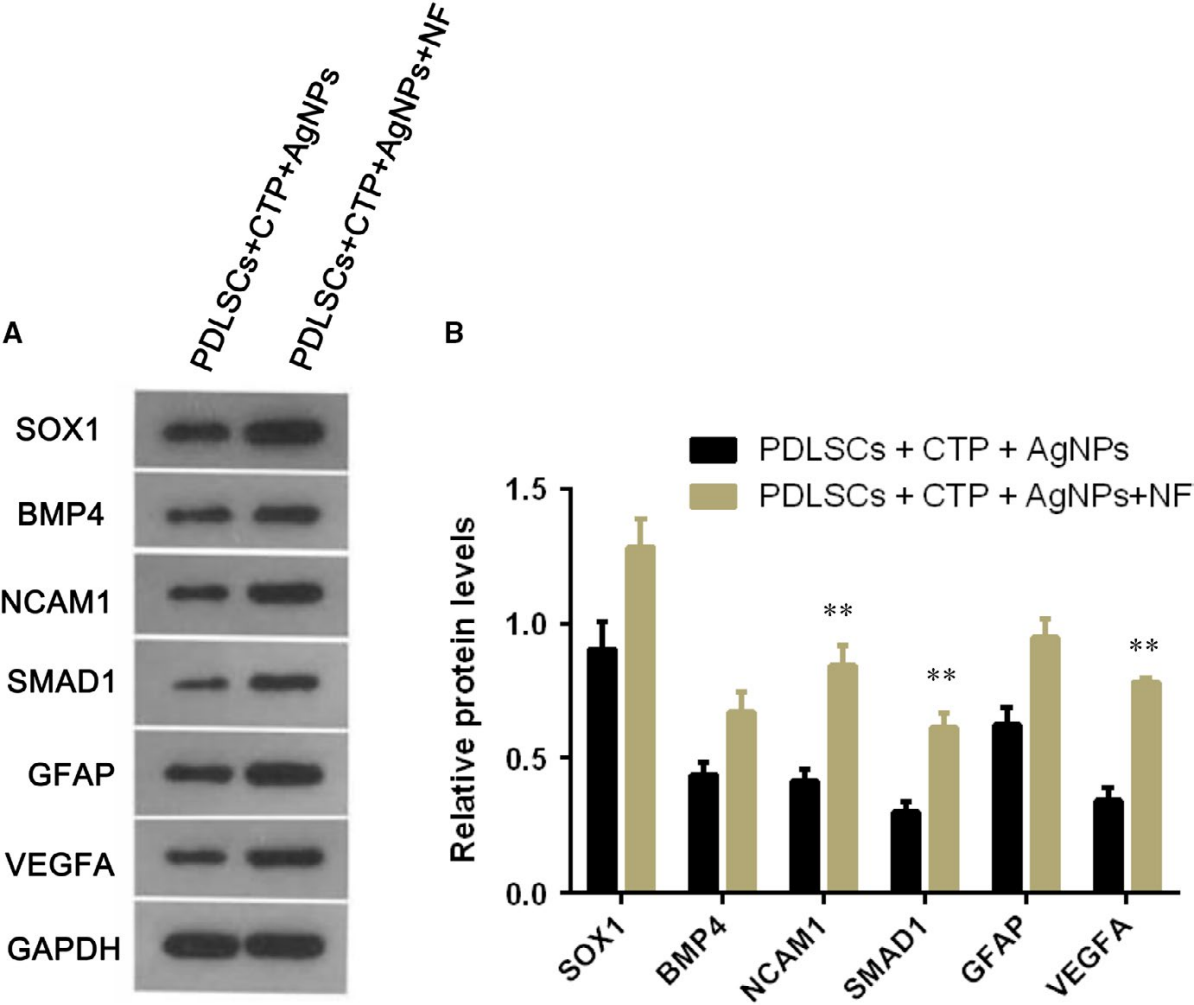
Western blot analysis showed proteins BMP4 and SMAD1 highly expressed in the PDLSCs induced odontogenesis‐like cells, while the neural cell‐associated markers, SOX1, NCAM1, SMAD1, GFAP and VEGFA highly expressed in the DPSCs induced neural cells (A). ^**^ *P* < .05 represented significant difference of proteins NCAM1, SMAD1 and VEGFA (B)

To explore the induced cell proliferation ability, the crystal violet assay and cell colony formation assay were all conducted, and results showed that both the odontogenesis‐like differentiated cells and neuronal differentiated cells were all with high proliferation ability from the third day to seventh day (Figure 3A); the cell viability curve of the seven days cultivated period was logarithmic growth trend (Figure 3B); moreover, the cell colony assay also identified the cells with high proliferation ability (Figure 4A), and the difference was significant in the seventh day compared with that in the third day (Figure 4B). Furthermore, the Western blot results revealed the CTP and AgNPs significantly induced the PDLSCs into odontogenesis‐like cell differentiation, for the proteins BMP4 and SMAD1, and were highly expressed. However, the L‐glutamine, NGF, bFGF, IGF‐1 and EGF growth factors administration promoted the proteins expression of neural cell‐associated markers, SOX1, NCAM1, SMAD1, GFAP and VEGFA; therefore, PDLSCs had the ability of neuronal cell differentiation (Figure 5).

## 4 DISCUSSION

From the study, we had provided the evidence that the calcined tooth powder (CTP) and silver nanoparticles (AgNPs) additives had the ability to induce the PDLSCs differentiate into odontogenesis, while the nutritional factor (L‐glutamine, NGF, bFGF, IGF‐1 and EGF) additives were prior to induce the PDLSCs into neuronal differentiation. The CTP ingredients were major tooth calcium powder and hyaluronic acid, which were suitable for the migration and osteogenic differentiation of dental stem cells^17^; besides, its’ good biological compatibility made it to be a useful therapy for dental regeneration.^18^ While the AgNps is effective for antibiotic‐resistant bacterial strains, which was able to accelerate the osteogenesis of urine‐derived stem cells through activating the RhoA signalling pathway.^19^ Additionally, the growth factor mixtures (NGF, bFGF, IGF‐1 and EGF) were benefit for the cell proliferation and differentiation^14^, ^15^, ^16^; hence, there is a possibility that the utility of PDLSCs for dental restoration could enhance the teeth sensitivity and perceptivity. In our study, with the cell crystal violet assay and clonogenic assay in vitro, results showed that the induced odontoblast and neural cells had high cell viability and proliferation ability, which further confirmed PDLSCs to be ideal candidates for teeth tissue regeneration. Moreover, the expression profiles of the induced odontoblast and neural cells were also detected, the immunofluorescence staining revealed an up‐regulated expression of the protein BMP4 with the CTP and AgNps additives supplemented, and the high dosage of CTP administration enhanced the odontoblast differentiation ability for the staining intensity of proteins BMP4 obviously increased. Besides, the proteins BMP4 and SMAD1 were highly expressed in the induced odontoblast, and the growth factors additives supplemented facilitated the PDLSCs into odontoblast differentiation with significantly up‐regulation of proteins BMP4 and SMAD1; studies of Lu Liu^20^ and H W Mi^21^ both found that protein BMP4 was involved in odontoblastic differentiation from periodontal ligament cells. In addition, Zhu S^22^ demonstrated ephrinB2‑EphB4 signalling pathway was important for odontoblastic differentiation with PDLSCs; hence, in our study, further studies are necessary to investigate the signalling pathway involved in odontoblastic differentiation of PDLSCs. While in the induced cells, the proteins SOX1, NCAM1, GFAP and VEGFA were all obviously expressed, this finding was consistent with the studies of Wada N,^23^ Ruiling Tang^24^ and Ng TK^25^; the three researchers all confirmed PDLSCs with the ability of osteogenic and neural differentiation. Therefore, PDLSCs had great potential in pulp therapy combined with regenerative medicine materials for their osteogenic differentiation and neural regeneration abilities.

However, this study had some shortcomings. These results were obtained from the in vitro circumstances and did not further confirmed in vivo. This would reduce the research value of this study. The complicated phenomenon in vivo such as the multiple cellular context would change the effects of PDLSCs in tissue engineering to a large extent. Maybe in the foreseeable future, much more evidences especially from the in vivo study would be further investigated and shed some new lights in regenerative medicine.

## CONFLICT OF INTEREST

All of the authors have no conflict of interest in this research.

## AUTHOR CONTRIBUTION

Xiaojie Li, Dapeng Liao conceived and designed the experiments, and performed the experiments; Xiaojie Li, Dapeng Liao,Gang Sun and HanWen Chu analysed the data; Gang Sun, HanWen Chu contributed reagents/materials/analysis tools.

## ETHICAL APPROVAL

The study was followed the Ethical legals of animals.

## Data Availability

The data are available if necessary.
